# Supplementary material for: CRISPR-Knockout of CSE Gene Improves Saccharification Efficiency by Reducing Lignin Content in Hybrid Poplar
Source: Int J Mol Sci. 2021 Sep 9;22(18):9750. doi: 10.3390/ijms22189750 (PMC8466951; doi:10.3390/ijms22189750)
Supplement: Supplementary file 1 [file ijms-22-09750-s001.zip › ijms-1367102-supplementary.pdf]

Supplementary materials

Table S1. Primers used in this study.

Gene ID	Gene Name	Primer direction	Sequence (5’-3’)
Constructs for producing transgenic plants			
Potri. 001G175000	*PtrCSE1*	Forward	ATGTCATCCGAAACGCAGCAACCC
		Reverse	TCAGTCATCGCTTTTTTTGGACC
Potri. 003G059200	*PtrCSE2*	Forward	ATGCCATCCGAAGCGCAGCA
		Reverse	TCAATCATCACTCTTTGTAGACC
For Mi-seq analysis			
Potri. 001G175000	*CSE1*-sg1_Mi-seq_1st	Forward	GTCAACCAGCCAAGCCT
		Reverse	GAGTTAATTACCCATGTAGCA
	CSE1-sg1_Mi-seq_2nd	Forward	ACACTCTTTCCCTACACGACGCTCTTCCGATCTATGTCATCCGAAACGCAGC
		Reverse	GTGACTGGAGTTCAGACGTGTGCTCTTCCGATCTGGTCATATATACCGTGGCTT
	CSE1-sg2_Mi-seq_1st	Forward	ATGGACAAGATTGCTGCCA
		Reverse	TCATCGCTTCTGGTATGACAA
	CSE1-sg2_Mi-seq_2nd	Forward	ACACTCTTTCCCTACACGACGCTCTTCCGATCTATGGACAAGATTGCTGCCA
		Reverse	GTGACTGGAGTTCAGACGTGTGCTCTTCCGATCTTCATCGCTTCTGGTATGACAA
Potri. 003G059200	*CSE2*-sg1_Mi-seq_1st	Forward	CTCTGTTTAAAAGTCAA
		Reverse	GTGCATTAATTACCCATGTAG
	CSE2-sg1_Mi-seq_2nd	Forward	ACACTCTTTCCCTACACGACGCTCTTCCGATCTATGCCATCCGAAGCGCA
		Reverse	GTGACTGGAGTTCAGACGTGTGCTCTTCCGATCTATACACCGTGGCTTTGACCT
	CSE2-sg3_Mi-seq_1st	Forward	CGACATGGAGAAAATTGCTGC
		Reverse	TCAATCATCACTCTTTGTAGACC
	CSE2-sg3_Mi-seq_2nd	Forward	ACACTCTTTCCCTACACGACGCTCTTCCGATCTCGACATGGAGAAAATTGCTGC
		Reverse	GTGACTGGAGTTCAGACGTGTGCTCTTCCGATCTATCACGCCCGTCCACGT
For quantitative RT-PCR			
Potri. 001G309500	*Poplar ACTIN7*	Forward	GCATCCACGAGACTACATACAACTCA
		Reverse	GTGATCTCCTTGCTCATTCGGTCA
Potri. 001G175000	*CSE1-sg2*	Forward	GCGCTTCAGCGAACCATACA
		Reverse	CATCGCTTCTGGTATGACAAAAAG
Potri. 003G059200	*CSE2-sg3*	Forward	GCCTTCTTATTTGGCGAGTC
		Reverse	TGCCTTACTAGGTTTCATTGGT
Potri. 010G224100.1	*PtrPAL4*	Forward	CCTACATTGACGATCCTTGCAG
		Reverse	GACCTGCATTCCTTGATCCTG
Potri. 006G033300.1	*PtrC3H3*	Forward	GAGGTTCCTGGAGGAGGATG
		Reverse	GGAGTCGTCATGTAAGTGAC
Potri. 013G157900.1	*PtrC4H1*	Forward	ACTCTGGGACGTCTGGTACA
		Reverse	GCTTCATAGATTTACAGTGACACCC

Potri. 019G130700.1	*PtrC4H2*	Forward	GAGCAAGATCCTGGTAAACGC
		Reverse	CTGAGGTGTCAATCTTGGACTG
Potri. 003G183900.1	*PtrHCT1*	Forward	ATCAGCATGTAAGGCACGCGG
		Reverse	TGCCAAAGTAACCAGGTGGAAGCGT
Potri. 003G188500.1	*Ptr4CL5*	Forward	CATCCGAGGTGATCAGATCATG
		Reverse	CACAGCAGCATCAGATATCC
Potri. 012G006400.1	*PtrCOMT2*	Forward	CATGAAGTGGATATGCCATG
		Reverse	GTTGAATGCACAGCACATTAC
Potri. 009G099800.1	*PtrCCOAoMT1*	Forward	CAAGAGGTTGATTGAGCTTG
		Reverse	GGTCAGCAGCAAGTGCCTTG
Potri. 003G181400.1	*PtrCCR2*	Forward	CTGTTCAAGCTTATGTGCATG
		Reverse	GTGGAGAACGCTCTCAGAGC
Potri. 009G095800.1	*PtrCAD1*	Forward	CAAGCTGATCTTGATGGGTG
		Reverse	CGAATCTATATCTCACATC
Potri. 001G118800.1	*PtrMYB92*	Forward	CGAATACTAACGACGACACG
		Reverse	GTGTTCCATCTCTAATGTGC
Potri. 017G130300.1	*PtrMYB152*	Forward	GAAGACTTGCTACTGCCAGAT
		Reverse	TCATTCTTGAGCACTGATTG

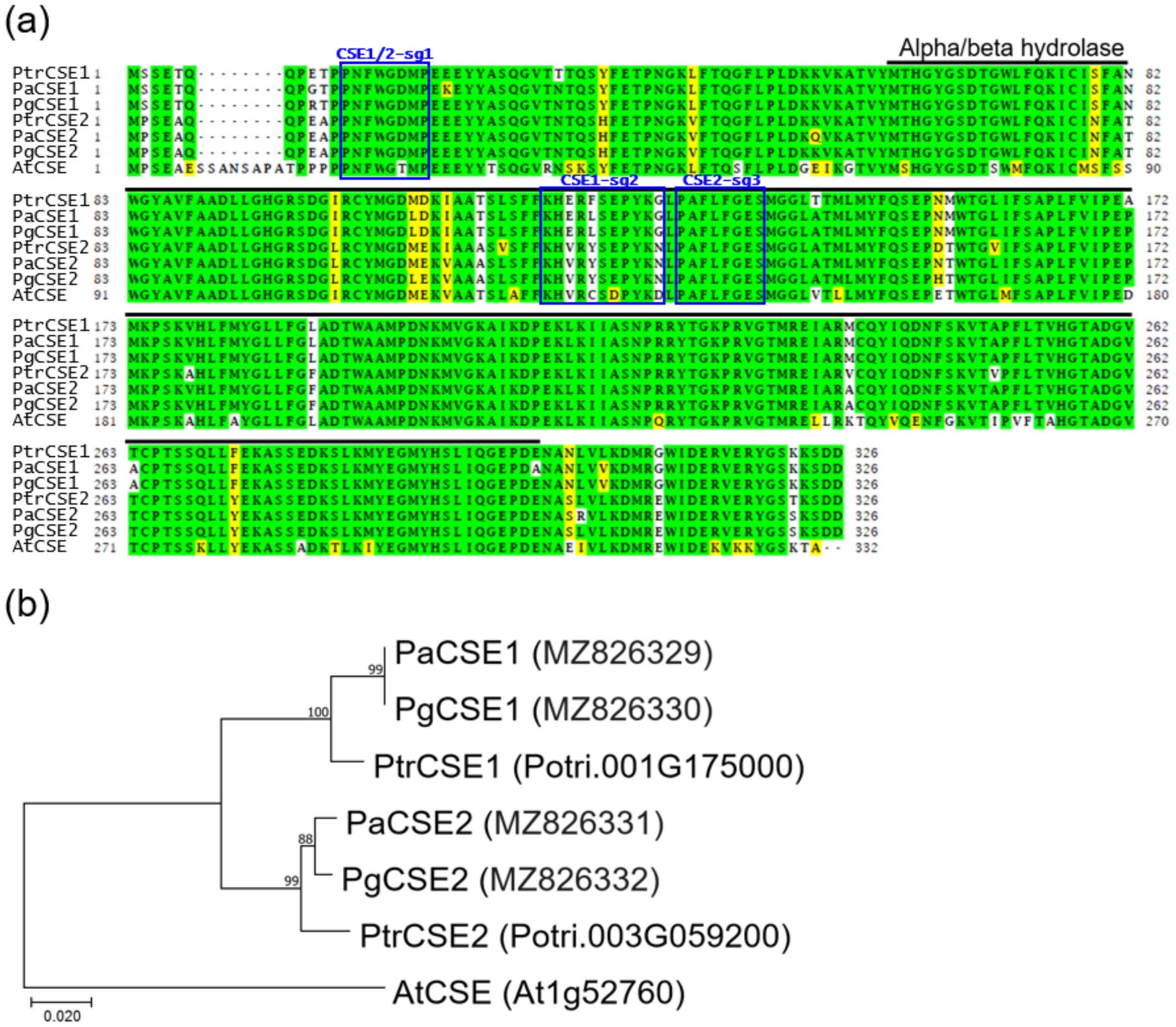

Figure S1. Sequence analysis of poplar *CSE* genes.

(a) Amino acid sequence alignment of *PtrCSE1* (Potri.001G175000), *PaCSE1* (MZ826329), *PgCSE1* (MZ826330), *PtrCSE2* (Potri.003G059200), *PaCSE2* (MZ826331), *PgCSE2* (MZ826332), and *Arabidopsis CSE* (At1g52760). Genbank accession numbers of *CSE* genes are shown in parenthesis. Blue boxes indicate the single guide RNA (sg1-sg3) target sequences and alpha/beta hydrolase domain is marked by black line. (b) Phylogenetic analysis of CSE proteins. Complete amino acid sequences were aligned using ClustalW and a rooted phylogenetic tree was constructed using MEGA 7 (https://www.megasoftware.net/) with the Neighbor-Joining method (1,000 bootstrap replicates) and Jones-Taylor-Thornton (JTT) model.

(a)

Single guide RNA / Target gene	#RGEN Target (5' to 3')	Position	Direction	GC Contents (w/o PAM)	Out-of-frame Score	Mismatches		
						0	1	2
Sg1 / *PaCSE1*	CAACTTCTGGGGCGACATGCCGG	Exon 1 36	+	60	65.3	2	0	0
PgCSE1	CAACTTCTGGGGCGACATGCCGG	36	+	60	63	2	0	0
PaCSE2	CAACTTCTGGGGCGACATGCCGG	36	+	60	58	2	0	0
PgCSE2	CAACTTCTGGGGCGACATGCCGG	36	+	60	58	2	0	0
Sg2 / *PaCSE1*	GCGCTTAAGCGAACCATACAAGG	Exon 2 366	+	50	57.7	0	1	0
PgCSE1	GCGCTTCAGCGAACCATACAAGG	366	+	55	54.9	1	0	0
Sg3 / *PaCSE2*	AACCTGCCCGCCTTCTTATTTGG	Exon 2 397	+	50	64.3	0	1	0
PgCSE2	AACCTGCCCGCCTTCTTATTTGG	397	+	50	64.3	0	1	0

(b)

Single guide RNA / Target gene	Target sequence
Sg1 / *PaCSE1/2* & *PgCSE1/2*	5'- **CCC**AACTTCTGGGGCGACATGCC -3'
Sg2 / *PaCSE1* & *PgCSE1*	5'- GCGTTAAGCGAACCATACA**AGG** -3'
Sg3 / *PaCSE2* & *PgCSE2*	5'- **CCC**GCCTTCTTATTTGGCGAGTC -3'

Figure S2. Design of single guide RNAs to target CSE genes in poplar.

(a) Design of single guide RNAs for CSE using Cas-Designer of the CRISPR RGEN Tools (http://www.rgenome.net/cas-designer). sg1 is the 37-56th nucleotide sequence of the first exon, and targets both *PagCSE1* and *PagCSE2*. sg2 and sg3 correspond to the 366-386th and 397-417th nucleotide sequences of the 2nd exons of *PagCSE1* and *PagCSE2*, respectively. Selected target sequences had a low number of mismatches and an out-of-frame score of 55 or higher. (b) Final single guide RNA sequences used in this study. The protospacer adjacent motif (PAM) sequence is underlined.
